# Prevalence and factors associated with difficulty and intention to quit smoking in Switzerland

**DOI:** 10.1186/1471-2458-11-227

**Published:** 2011-04-13

**Authors:** Pedro Marques-Vidal, João Melich-Cerveira, Fred Paccaud, Gérard Waeber, Peter Vollenweider, Jacques Cornuz

**Affiliations:** 1 Institute of Social and Preventive Medicine (IUMSP), Centre Hospitalier Universitaire Vaudois and University of Lausanne, Bugnon 17, 1005 Lausanne, Switzerland; 2 Clinical Trials Unit, University Hospital (CHUV), Lausanne, Switzerland; 3 Medical Faculty of the University of Lisbon, Lisbon, Portugal; 4 Department of Medicine, University Hospital (CHUV), Lausanne, Switzerland; 5 Department of Ambulatory Care and Community Medicine, University of Lausanne, Bugnon 44, 1011 Lausanne, Switzerland

## Abstract

**Background:**

recent data indicate a slight decrease in the prevalence of smoking in Switzerland, but little is known regarding the intention and difficulty to quit smoking among current smokers. Hence, we aimed to quantify the difficulty and intention to quit smoking among current smokers in Switzerland.

**Methods:**

cross-sectional study including 607 female and 658 male smokers. Difficulty, intention and motivation to quit smoking were assessed by questionnaire.

**Results:**

90% of women and 85% of men reported being "very difficult" or "difficult" to quit smoking. Almost three quarters of smokers (73% of women and 71% of men) intended to quit; however, less than 20% of them were in the preparation stage and 40% were in the precontemplation stage. On multivariate analysis, difficulty to quit was lower among men (Odds ratio and 95% [confidence interval]: 0.51 [0.35-0.74]) and increased with nicotine dependence and number of previous quitting attempts (OR = 3.14 [1.75 - 5.63] for 6+ attempts compared to none). Intention to quit decreased with increasing age (OR = 0.48 [0.30-0.75] for ≥65 years compared to < 45 years) and increased with nicotine dependence, the number of previous quitting attempts (OR = 4.35 [2.76 - 6.83] for 6+ attempts compared to none) and among non-cigarette smokers (OR = 0.51 [0.28 - 0.92]). Motivation to quit was inversely associated with nicotine dependence and positively associated with the number of previous quitting attempts and personal history of lung disease.

**Conclusion:**

over two thirds of Swiss smokers want to quit. However, only a small fraction wishes to do so in the short term. Nicotine dependence, previous attempts to quit or previous history of lung disease are independently associated with difficulty and intention to quit.

## Background

Cigarette smoking is the most important modifiable risk factor for premature death in the world causing 5.4 million deaths every year [1], and it is expected that by 2030 an estimated 7.4 to 9.7 million deaths will be attributable to tobacco smoking [1]. In Switzerland, health costs related to smoking have been estimated at 10 billion CHF a year [2]. The Swiss Federal Office of Public Health has launched comprehensive tobacco prevention program focusing on specific interventions and cooperation between partners for tobacco prevention [3], and recent data indicate a slight decrease in the prevalence of smoking [4] but little is known regarding the intention and difficulty to quit smoking among current smokers in Switzerland [5].

Hence, we used the data from a large, population-based, cross-sectional study (CoLaus study) to assess the prevalence and clinical factors related to intention and difficulty to quit smoking among Swiss smokers. The population of Lausanne (from which the CoLaus study is drawn) can be considered as representative of the whole country as a considerable proportion of the Lausanne population is non-Swiss or comes from other cantons: in 2006, out of the 128,231 Lausanne inhabitants, 38% were non-Swiss, 30% came from other cantons (including Italian and German-speaking cantons) and only 32% were actually from the Vaud canton [6].

## Methods

### Recruitment process and inclusion criteria

This study was focused on current smokers from the CoLaus study, the design of which has been previously described [7]. Briefly, it is a population-based study conducted between 2003 and 2006 which recruited over 6,000 subjects aged 35-75 years in Lausanne, Switzerland. The following inclusion criteria were applied: a) voluntary participation to the examination, including blood sample, b) aged 35-75 years, and c) Caucasian origin defined as having both parents and grand-parents Caucasian (determined by birth place). The Institutional Review Board of the Centre Hospitalier Universitaire Vaudois (CHUV) in Lausanne and the Cantonal Ethics Committee approved the study protocol and signed informed consent was obtained from participants. Participants were asked to attend the outpatient clinic at the CHUV, Lausanne, in the morning after an overnight fast. Data were collected by trained field interviewers during a single visit lasting about 60 minutes. Overall participation rate was 41%.

### Smoking status

The amount of tobacco smoked (number of cigarettes, cigarillos, cigars, or pipes per day) was asked and converted into cigarette equivalents. Cigarette equivalents were assessed as described previously [8]: 1 cigarillo or 1 pipe = 2.5 cigarettes; 1 cigar = 5 cigarettes. Smokers who consumed tobacco products other than cigarettes were considered as non-cigarette smokers. Nicotine dependence was assessed by the heaviness of smoking index (HSI) [9]. The HSI is the sum of two categorical measures: number of cigarettes smoked per day (codes 0:0-10 cigarettes per day; 1: 11-20; 2: 21-30; 3: 31+), and time to first cigarette after waking (coded: 0: 61+ minutes; 1: 31-60 min; 2: 6-30 min; 3: 5 min or less). Values for HSI range from 0 to 6.

Difficulty and intention to quit smoking were addressed by questionnaire. The questions included a) difficulty to stop smoking (4 possible answers: "very difficult", "difficult", "easy" and "very easy"; difficulty was considered for the first two answers); b) the number of attempts to quit smoking during the last 12 months and c) intention to quit smoking (4 possible answers: "yes, definitely", "yes"; "no" and "not at all"; intention to quit smoking was considered for the first two answers).

If participants intended to quit smoking, motivation to quit was measured using a modified algorithm [10] of the state of change construct as suggested previously [11] and applied in Switzerland [12]. Briefly, the groups were defined as follows: Precontemplation, not interested in quitting smoking in next 6 months; Contemplation, interested in quitting smoking in next 6 months but not next 30 days; Preparation, interested in quitting smoking in next 30 days.

### Other data

Information on demographic data, socio-economic and marital status, lifestyle factors, personal and family history of cardiovascular and lung disease (defined as emphysema and/or chronic bronchitis) was collected. Educational level was categorized into basic, apprenticeship, secondary and university. Participants were then categorized as drinkers if they had consumed at least one alcohol drink the last seven days and as non-drinkers otherwise. Subjects were also categorized according to their level of alcohol consumption: none, low (1-6 drinks/week); moderate (7-13 drinks/week), high (14-34 drinks/week) and very high (≥35 drinks/week) [13]. Participants were considered as physically active if they reported practicing at least 2 hours of leisure-time physical activity per week.

### Statistical analysis

Statistical analyses were conducted using Stata v10.1 (Stata Corp, Texas, USA) and SAS v9.2 (SAS, Cary, USA) for Windows. As the number of quitting attempts during the last 12 months was considerably skewed, three groups were made: no attempt, < 6 attempts and ≥6 attempts. Also, HIS values were categorized into low (0-1), medium (2-4) and high (5-6) as suggested [14]. Results were expressed as mean ± standard deviation or as number of participants and (percentage). Bivariate comparisons were performed using the Student's t-test or chi-square test for continuous and discrete variables, respectively. The clinical factors independently and significantly related with difficulty and intention to quit were assessed by stepwise multivariate logistic regression. Statistical significance was considered for p < 0.05.

## Results

### Characteristics of the sample

Overall, 1385 current smokers were invited to participate, of which 1265 (91%) responded and 1234 (89%) provided complete information on difficulty and intention to quit. The characteristics of the 1234 participants according to gender are summarized in table 1. Men were more educated, more frequently married but less active than women; men also reported a higher frequency of alcohol consumption and of personal history of coronary heart disease than women. Men smoked more and had higher nicotine dependence (assessed by HSI) than women. Men also tended to smoke more frequently tobacco products other than cigarettes, while no differences were found for the number of quitting attempts (table 1). Finally, 19.6% of the participants smoked within 5 minutes after waking up, and 51.4% within 30 minutes, no differences being found between genders (not shown).

**Table 1:** participants' characteristics, overall and by gender

	All (N = 1234)	Women (N = 591)	Men (N = 643)	Test
Age (years)	50.9 ± 9.9	51.2 ± 9.6	50.5 ± 10.1	1.35 ^NS^
Married (%)	609 (49.4)	245 (41.5)	364 (56.6)	28.3 ***
Educational level (%)				
Basic	289 (23.4)	138 (23.4)	151 (23.5)	10.46 *
Apprenticeship	493 (40.0)	236 (39.9)	257 (40.0)	
Secondary	269 (21.8)	146 (24.7)	123 (19.1)	
University	183 (14.8)	71 (12.0)	112 (17.4)	
Leisure-time physical activity (%)	554 (44.9)	288 (48.7)	266 (41.4)	6.74 **
Alcohol drinker (%)	958 (77.6)	403 (68.2)	555 (86.3)	58.3 ***
Alcohol consumption (%)				
None	276 (22.4)	188 (31.8)	88 (13.7)	180 ***
1-6/week	374 (30.3)	228 (38.6)	146 (22.7)	
7-13/week	238 (19.3)	104 (17.6)	134 (20.8)	
14-34/week	297 (24.0)	67 (11.3)	230 (35.8)	
35+/week	49 (4.0)	4 (0.7)	45 (7.0)	
Personal history of (%)				
CVD	66 (5.3)	19 (3.2)	47 (7.3)	10.2 ***
Lung disease	107 (8.7)	51 (8.6)	56 (8.7)	0.01 ^NS^
Family history of (%)				
CHD	364 (29.5)	187 (31.6)	177 (27.5)	2.51 ^NS^
Lung disease	106 (8.6)	59 (10.0)	47 (7.3)	2.80 ^NS^
Smoking amount ^a^	18.2 ± 12.5	16.0 ± 10.4	20.2 ± 13.8	6.07 ***
Smoking amount ^a ^(%)				
1-4/day	115 (9.3)	62 (10.5)	53 (8.2)	31.4 ***
5-14/day	332 (26.9)	192 (32.5)	140 (21.8)	
15-24/day	515 (41.8)	247 (41.9)	268 (41.7)	
25+/day	271 (22.0)	89 (15.1)	182 (28.3)	
Non-cigarette smokers (%)	55 (4.5)	7 (1.2)	48 (7.5)	28.5 ***
Heaviness of smoking index	2.4 ± 1.8	2.2 ± 1.8	2.6 ± 1.8	3.33 ***
Nicotine dependence ^b ^(%)				
Low (0-1)	437 (35.4)	228 (38.6)	209 (32.5)	14.24 ***
Medium (2-4)	629 (51.0)	304 (51.4)	325 (50.5)	
High (5-6)	168 (13.6)	59 (10.0)	109 (17.0)	
Number of quitting attempts (%)				
None	412 (33.4)	183 (31.0)	229 (35.6)	5.51 ^NS^
1-5	621 (50.3)	318 (53.8)	303 (47.1)	
6+	201 (16.3)	90 (15.2)	111 (17.3)	

Results are expressed as number of subjects and (percentage) or as mean ± standard deviation. ^a ^in cigarette equivalents; ^b^, assessed by the heaviness of smoking index. CHD, coronary artery disease; CVD, cardiovascular disease. Statistical analysis by chi-square or Student's t-test comparing women and men: ^NS^, not significant; *, p < 0.05; **, p < 0.01; ***, p < 0.001.

### Factors associated with difficulty and intention to quit

Almost nine out of ten smokers reported difficulty quitting smoking, and two thirds intended to quit. The factors associated with difficulty and intention to quit smoking are summarized in table 2. Difficulty to quit was significantly and positively associated with female gender, personal and family history of lung disease, nicotine dependence (as assessed by heaviness of smoking index) and number of attempts to quit, and negatively associated with leisure-time physical activity. Conversely, no association was found with marital status, education, leisure-time physical activity, alcohol drinker, personal history of coronary heart disease and family history of cardiovascular or lung disease (not shown). Intention to quit was significantly and positively associated with nicotine dependence, number of attempts to quit and personal and family history of lung disease, while it was negatively associated with age and alcohol consumption.

**Table 2:** factors associated with difficulty and intention to quit smoking

	N	Difficulty	Test	Intention	Test
All	1234	1075 (87.1)		883 (71.6)	
Gender					
Women	591	530 (89.7)	6.64 **	429 (72.6)	0.59 ^NS^
Men	643	545 (84.8)		454 (70.6)	
Age group					
[35-44]	416	360 (86.5)	3.37 ^NS^	314 (75.5)	20.9 ***
[45-54]	397	355 (89.4)		300 (75.6)	
[55-64]	310	267 (86.1)		210 (67.7)	
[65+]	111	93 (83.8)		62 (55.9)	
Leisure-time physical activity					
No	680	608 (89.4)	7.12 **	488 (71.8)	0.03 ^NS^
Yes	554	487 (87.9)		395 (71.3)	
Alcohol drinker					
No	276	247 (89.5)	1.79 ^NS^	213 (77.2)	5.51 *
Yes	958	828 (86.4)		670 (69.9)	
Alcohol consumption (%)					
None	276	247 (89.5)	2.55 ^NS^	213 (77.2)	6.34 ^NS^
1-6/week	374	325 (86.9)		266 (71.1)	
7-13/week	238	204 (85.7)		165 (69.3)	
14-34/week	297	255 (85.9)		207 (69.7)	
35+/week	49	44 (89.8)		32 (65.3)	
Personal history of lung disease					
No	1127	971 (86.2)	10.6 ***	794 (70.5)	7.77 **
Yes	107	104 (97.2)		89 (83.2)	
Family history of lung disease					
No	1128	974 (86.3)	6.89 **	797 (70.7)	5.22 *
Yes	106	101 (95.3)		86 (81.1)	
Non-cigarette smokers					
No	1179	1038 (88.0)	20.2 ***	858 (72.8)	19.3 ***
Yes	55	37 (67.3)		25 (45.5)	
Nicotine dependence ^a^					
Low (0-1)	437	308 (70.5)	168.7 ***	271 (62.0)	31.2 ***
Medium (2-4)	629	600 (95.4)		478 (76.0)	
High (5-6)	168	167 (99.4)		134 (79.8)	
Number of quitting attempts					
None	412	326 (79.1)	35.2 ***	238 (57.8)	65.3 ***
1-5	621	565 (91.0)		472 (76.0)	
6+	201	184 (91.5)		173 (86.1)	

Results are expressed as number of subjects and (row percentage). Bivariate statistical analysis by chi-square: ^NS^, not significant; *, p < 0.05; **, p < 0.01; ***, p < 0.001. ^a ^assessed by the heaviness of smoking index.

Stepwise multivariate logistic regression was applied to identify the variables significantly associated with difficulty and intention to quitting smoking, and the results are summarized in table 3. Difficulty to quit was lower among men and increased with increasing nicotine dependence and the number of previous quitting attempts. Intention to quit decreased with increasing age and increased with nicotine dependence, the number of previous quitting attempts and among non-cigarette smokers (table 3).

**Table 3:** multivariate analysis of the factors associated with difficulty, intention and motivation to quit smoking

	Difficulty	Intention	Preparation ^a^
Gender			
Women	1 (ref.)	-	-
Men	0.51 [0.35 - 0.74]	-	-
Age group			
[35-44]	-	1 (ref.)	-
[45-54]	-	0.98 [0.71 - 1.37]	-
[55-64]	-	0.73 [0.52 - 1.02]	-
[65+]	-	0.54 [0.34 - 0.85]	-
Personal history of lung disease			
No	-	-	1 (ref.)
Yes	-	-	2.15 [1.30 - 3.57]
Non-cigarette smoker			
No	-	1 (ref.)	-
Yes	-	0.51 [0.28 - 0.92]	-
Nicotine dependence ^b^			
Low (0-1)	1 (ref.)	1 (ref.)	1 (ref.)
Medium (2-4)	8.67 [5.61 - 13.4]	1.75 [1.32 - 2.32]	0.62 [0.43 - 0.89]
High (5-6)	82.3 [11.3 -597.0]	2.32 [1.49 - 3.59]	0.53 [0.30 - 0.95]
Number of quitting attempts			
None	1 (ref.)	1 (ref.)	1 (ref.)
1 to 5	2.03 [1.36 - 3.02]	2.02 [1.53 - 2.66]	2.22 [1.42 - 3.47]
6+	3.14 [1.75 - 5.63]	4.39 [2.79 - 6.91]	5.18 [3.17 - 8.47]

Results are expressed as multivariate-adjusted odds ratio and [95% confidence interval]. ^a ^odds ratios for preparation stage vs. precontemplation + contemplation; ^b ^assessed by the heaviness of smoking index. Multivariate statistical analysis by stepwise logistic regression: -, not retained in the model. Other variables initially introduced in the model but not retained: marital status, education, leisure-time physical activity, alcohol drinker, personal history of coronary heart disease and family history of cardiovascular or lung disease.

### Factors associated with motivation to quit

Motivation to quit was assessed in the 883 subjects who indicated they wanted to quit (429 women and 454 men). Slightly less than one-fifth (19.5%) of them was in the preparation stage and four out of ten in the precontemplation stage. The factors associated with motivation to quit are summarized in table 4. Nicotine dependence was associated with a lower motivation to quit, while the number of previous quitting attempts and being physically active were positively associated. Conversely, no differences in motivation were found for gender, age group, alcohol consumption or personal/family history of lung disease.

**Table 4:** factors associated with motivation to quit smoking

	N	Precontemplation	Contemplation	Preparation	Test
All	883	353 (40.0)	358 (40.5)	172 (19.5)	
Gender					
Women	429	177 (41.3)	173 (40.3)	79 (18.4)	0.84 ^NS^
Men	454	176 (38.8)	185 (40.7)	93 (20.5)	
Age group					
[35-44]	314	124 (39.9)	121 (38.9)	66 (21.2)	2.85 ^NS^
[45-54]	300	116 (38.7)	125 (41.7)	59 (19.6)	
[55-64]	210	85 (40.5)	86 (40.9)	39 (18.6)	
[65+]	62	28 (45.2)	26 (41.9)	8 (12.9)	
Leisure-time physical activity					
No	488	211 (43.2)	193 (39.6)	84 (17.2)	6.04 *
Yes	395	142 (36.0)	165 (41.7)	88 (22.3)	
Alcohol drinker					
No	213	85 (40.0)	90 (42.2)	38 (17.8)	0.59 ^NS^
Yes	670	268 (40.0)	268 (40.0)	134 (20.0)	
Alcohol consumption					
None	214	85 (39.7)	91 (42.5)	38 (17.8)	3.38 ^NS^
1-6/week	270	100 (37.0)	122 (41.5)	58 (21.5)	
7-13/week	167	70 (41.9)	67 (40.1)	30 (18.0)	
14-34/week	211	88 (41.7)	80 (37.9)	43 (20.4)	
35+/week	32	15 (46.9)	11 (34.4)	6 (18.7)	
Personal history of lung disease					
No	794	325 (40.9)	322 (40.6)	147 (18.5)	5.56 ^NS^
Yes	89	28 (31.5)	36 (40.5)	25 (28.0)	
Family history of lung disease					
No	797	326 (40.9)	316 (39.6)	155 (19.5)	3.38 ^NS^
Yes	86	27 (31.4)	42 (48.8)	17 (19.8)	
Non-cigarette smokers					
No	858	343 (40.0)	347 (40.4)	168 (19.6)	0.24 ^NS^
Yes	25	10 (40.0)	11 (44.0)	4 (16.0)	
Nicotine dependence ^a^					
Low (0-1)	271	101 (37.3)	96 (35.4)	74 (27.3)	16.7 **
Medium (2-4)	478	192 (40.2)	206 (43.1)	80 (16.7)	
High (5-6)	134	60 (44.8)	56 (41.8)	18 (13.4)	
Number of quitting attempts					
None	238	130 (54.6)	79 (33.2)	29 (12.2)	48.5 ***
1-5	472	175 (37.1)	211 (44.7)	86 (18.2)	
6+	173	48 (27.7)	68 (39.3)	57 (33.0)	

Results are expressed as number of subjects and (row percentage). Bivariate statistical analysis by chi-square: ^NS^, not significant; *, p < 0.05; **, p < 0.01; ***, p < 0.001. ^a ^assessed by the heaviness of smoking index.

Stepwise multivariate logistic regression was applied to identify the variables significantly associated with being in the preparation stage, and the results are summarized in table 3. Nicotine dependence was negatively associated while number of quitting attempts and personal history of lung disease were positively associated with a higher motivation.

### Comparison with non-responders

The comparison of responders with non-responders is summarized in table 5. Non-responders were better educated, had a higher prevalence of alcohol consumption and physical activity and a lower frequency of family history of lung disease. Non-responders also smoked considerably less than responders.

**Table 5:** comparison between responders and non responders

	Non responders (N = 151)	Responders (N = 1234)	Test
Women (%)	69 (45.7)	591 (47.9)	0.26 ^NS^
Married (%)	80 (53.0)	609 (49.4)	0.71 ^NS^
Age (years)	52.3 ± 10.7	50.9 ± 9.9	1.72 ^NS^
Educational level (%)			
Basic	24 (15.9)	289 (23.4)	
Apprenticeship	48 (31.8)	493 (40.0)	20.1 ***
Secondary	37 (24.5)	269 (21.8)	
University	42 (27.8)	183 (14.8)	
Leisure-time physical activity (%)	95 (62.9)	554 (44.9)	17.5 ***
Alcohol drinker (%)	128 (84.8)	958 (77.6)	4.05 *
Personal history of (%)			
CVD	9 (6.0)	66 (5.3)	0.10 ^NS^
Lung disease	6 (4.0)	107 (8.7)	3.96 *
Family history of (%)			
CHD	42 (27.8)	364 (29.5)	0.18 ^NS^
Lung disease	6 (4.0)	106 (8.6)	3.86 *
Smoking amount ^a^	7.1 ± 10.2	18.2 ± 12.5	10.5 ***

Results are expressed as number of subjects and (percentage) or as mean ± standard deviation. ^a ^in cigarette equivalents. Statistical analysis by chi-square, Fisher's exact test or Student's t-test: ^NS^, not significant; *, p < 0.05; **, p < 0.01; ***, p < 0.001.

## Discussion

There is few data regarding intention to quit smoking in Switzerland. This cross-sectional, population-based study thus provides important information regarding the current intention to quit among current smokers, as well as the associated clinical factors. Furthermore, the ongoing follow-up of the entire CoLaus cohort will enable a better assessment of the factors associated with successful quitting.

Apprentices had a higher smoking prevalence. These findings are in agreement with the literature [15] and might be related to the fact that, compared to their high school counterparts, apprentices receive a monthly wage, are in contact with more adults and are not systematically submitted to smoking bans in their working places, thus making them more prone to start smoking.

Nicotine dependence, assessed by HSI, smoking amount or the percentage of subjects who smoked within 5 minutes after waking up, was within the values reported in the literature. For instance, almost one out of five smokers smoked within 5 minutes after waking, a figure comparable to Poland, the UK or Sweden [16], but lower than Greece [16] and considerably higher than Germany [16] or than previous Swiss studies [5]. Overall, our data indicate that the level of nicotine dependence among smokers in Switzerland is comparable to other countries. The higher nicotine dependence found in this study compared to other Swiss studies might be related to sampling issues, as subjects from other studies were recruited by phone, and it has been suggested that phone surveys tend to underestimate the number of heavy smokers [17].

Almost nine out of ten smokers reported difficulty quitting smoking. In agreement with the literature [18], men reported a lower difficulty quitting smoking. Possible explanations for the increased difficulty in quitting smoking by women are the fear of weight gain after smoking cessation [19] and increased nicotine dependency in women [20], although the last possibility is not substantiated in this study. Increased nicotine dependence and the number of previous attempts to quit were also independently related with difficulty in quitting smoking. As smoking prevalence and smoking amount have been decreasing in Switzerland [4], it is likely that support measures aimed at quitting smoking will become more successful in the future.

Smoking cessation is positively associated with non-cigarette smoking [21], possibly due to the fact that this group may represent smokers in the process of transition to non-smoking. Indeed, in this study, non-cigarette smokers reported a lower difficulty in quitting smoking, but reported also a lower intention to quit, which was further confirmed on multivariate analysis. Possible explanations include their self-reported lower difficulty to quit or the false belief that non-cigarette smoking is less harmful than cigarette smoking, but further studies are needed to better assess this point.

Almost 72% of smokers wished to quit. This value is considerably higher than previously reported for Switzerland [5,22,23], Greece [24], the UK [25], Finland [26] and France [27], lower than Canada [25] and comparable to Australia, the USA [25] and other countries [16]. This high value might be due to the higher nicotine dependence among the study participants as indicated previously. Indeed, nicotine dependence was independently and positively related with intention to quit, a finding in agreement with the literature [14,28]. These findings suggest that heavy smokers are aware of their nicotine dependence and that adequate support should be provided. Similarly, the number of previous quitting attempts was positively related with intention to quit. These findings are in agreement with the literature [29] and suggest that several failures, known to cause frustration and loss of self-esteem in smokers [30], do not deter them from quitting smoking and that support should be provided irrespective of the number of previous quitting attempts. In agreement with the literature [27], personal history of lung disease was also positively related with intention to quit. In fact, health concern is the most frequently mentioned reason for abandoning tobacco consumption found in the literature [31]. Nevertheless, from a Public Health point of view, it would be better to motivate smokers to quit before serious smoking-related health problems arise. Conversely, the fact that old smokers do not wish to stop smoking is in agreement with some literature [25,32] but not with other [24,33] and might be associated with a more tolerant environment, namely if relatives or friends also smoke [18]. Another possible explanation is that elderly smokers do not perceive any benefit from quitting at an advanced age [34] or perceive themselves as less vulnerable to the harms of smoking [35]. Hence, our results indicate that efforts aimed at helping smokers to quit should be started at an early age as the older the smoker, the less likely he/she intended to quit; nevertheless, interventions adequately tailored to elderly smokers also achieve significant quitting rates [36]. Finally, no independent association was found between intention to quit smoking and alcohol consumption, marital status or education. Those findings are in agreement with some studies [24] but not with others [25,26,37,38] and suggest that family environment, alcohol consumption or educational level might exert a lower effect on intention to quit than age, nicotine dependence and personal history of lung disease.

A considerable percentage of smokers (29%, corresponding to 40.5% of smokers intending to quit) was in the contemplation state, a value higher than previously reported for Switzerland [5,22,23], Germany [39] and other countries [32], but within or even below values reported for Canada [40]. Also, circa 14% of smokers (19.5% of smokers intending to quit) were in the preparation state, a value higher than previously reported for Switzerland [5] but lower than in Canada [40]. This rise in the percentage of smokers in the contemplation and preparation states can be explained by the rising public awareness in Switzerland concerning tobacco-related illness and suggest that Swiss smokers are slightly more motivated to quit than in other countries, although further improvements are still achievable. As for intention to quit, nicotine dependency was negatively related and previous quitting attempts were positively related with motivation to quit, while no significant independent relationships were found with gender, age, marital status and alcohol consumption. Overall, our results indicate that the factors that or which influence intention to quit also influence the motivation of quitters; namely, previous unsuccessful quitting attempts do not appear to de-motivate smokers from quitting. Our results also suggest that physical activity might be a good adjunct to promote quitting smoking and smoking abstinence, perhaps by improving mood and self-efficacy [41]. However, further studies are needed, as physical activity was no longer related with motivation on multivariate analysis.

This study has some limitations. First, the overall participation rate of the CoLaus study was low (41%), which might limit the generalization of the findings; however, this participation rate is similar to other epidemiological studies [42]. Further, not all smokers answered the questionnaire and significant differences were found between responders and non-responders regarding age, personal or family history of lung disease and amount of tobacco smoked (table 5). Further, comparing the CoLaus data with the data from the Swiss Health Surveys from 2002 and 2007 for the same age groups showed a lower prevalence of current smokers and a higher prevalence of former smokers in our study (table 6), although the differences were less strong when compared with data from the same canton (table 6). Hence, it is possible that our study includes more health-conscious participants, which might overestimate rates of willingness to quit and underestimate the rates of difficulty to quit. Still, in the absence of other studies, our results provide a first estimation of the willingness and motivation to quit smoking in Switzerland. For instance, the National Survey on Tobacco indicates that 34% of current smokers have been advised to quit, but no information regarding individual willingness and motivation to quit smoking was collected [43]. Also, state of change might not distinguish properly (pre)contemplators [44] and it has been suggested that individual components should be used instead [45]. Still, similar findings were obtained when using intention to quit within 30 days or within 6 months instead of the state of change, or when using overall tobacco consumption or time between waking up and first cigarette instead of HSI (not shown). Only subjects of Caucasian origin were included in this study, and inference should be done accordingly, as genetic differences regarding intention and easiness to quit smoking may exist [46]. Further, some studies [47-49] but not others [50] suggest that alcohol dependent individuals have more difficulty to quit smoking than drinkers without alcohol dependence. However, the methods of the present study did not allow us to measure adequately alcohol dependence and no clear association was found with increased alcohol consumption. Also, no information was available regarding unemployment status, a factor known to be associated with increased smoking [51,52] and decreased quitting rates [53,54], which would represent a particular target group for health promotion or smoking cessation. Finally, as Switzerland has been recently placed in stage 4 of the smoking epidemic model, a stage characterized by the peak of social awareness concerning the hazards of tobacco smoking [55], the rise in the percentage of smokers wishing to quit may simply be a reflection of this rise in public consciousness.

**Table 6:** comparison of smoking rates between the CoLaus study and the Swiss Health Surveys 2002-7, overall and for the Vaud canton

	SHS (Switzerland)	CoLaus	SHS (VD canton)
**Men (N)**	**11,703**	**2,935**	**707**
Never	37.3 [36.4 - 38.2]	32.2 [30.5 - 33.9]	34.7 [31.1 - 38.3]
Former	29.7 [28.8 - 30.5]	38.5 [36.7 - 40.3]	32.5 [29.1 - 36.1]
Current	33.0 [32.2 - 33.9]	29.3 [27.7 - 31.0]	32.8 [29.4 - 36.4]
**Women (N)**	**14,049**	**3,251**	**895**
Never	53.6 [52.7 - 54.4]	47.2 [45.5 - 48.9]	51.1 [47.7 - 54.4]
Former	21.0 [20.4 - 21.7]	27.8 [26.3 - 29.4]	24.8 [22.0 - 27.8]
Current	25.4 [24.7 - 26.1]	25.0 [23.5 - 26.5]	24.1 [21.4 - 27.1]

Results are expressed as percentage and [95% confidence interval].

## Conclusion

In summary, our results indicate that two thirds of Swiss smokers want to quit, but only a small fraction is in the preparation state. Nicotine dependence, previous attempts to quit, physical activity or previous history of lung disease are independently associated with difficulty and intention to quit.

## Competing interests

The authors declare that they have no competing interests.

## Authors' contributions

PMV and JMC analysed the data, drafted and revised the paper. GW and PV initiated the CoLaus study, monitored data collection for the whole study and revised the paper. FP and JC monitored data collection and revised the draft paper. All authors read and approved the final manuscript.

## Pre-publication history

The pre-publication history for this paper can be accessed here:

http://www.biomedcentral.com/1471-2458/11/227/prepub
